# Supplementary material for: Neutralization of zoonotic retroviruses by human antibodies: Genotype-specific epitopes within the receptor-binding domain from simian foamy virus
Source: PLoS Pathog. 2023 Apr 24;19(4):e1011339. doi: 10.1371/journal.ppat.1011339 (PMC10159361; doi:10.1371/journal.ppat.1011339)
Supplement: S1 Table — (DOCX) [file ppat.1011339.s001.docx]

## S1 Table. SFV Env proteins produced for the study

| Name | Description^a^ | Expression level^b^ | Coomassie gel shown in Supplementary Fig. 4 | Neutralization experiments presented in | Comment |
| --- | --- | --- | --- | --- | --- |
| **Immunoadhesins** | (SU fused to murine Fc and Strep-tag |  |  |  |  |
| ^CI^SU | WT immunoadhesin, CI-PFV strain |  | Panel A | Fig. 7 |  |
| Kifu ^CI^SU | WT immunoadhesin produced in the presence of Kifunensine | Normal | Panel B | Fig. 7 |  |
| Kifu+Endo-H ^CI^SU | WT immunoadhesins produced in the presence of Kifunensine and treated with Endo-H | Normal | Panel B | Fig. 7 |  |
| ^CI^ΔN10 | N423>A | Normal | Panel E | Fig. 7 |  |
| ^CI^ΔRBDj | ΔF397-S483 | Normal | Panel E | Fig. 7 |  |
| ^CI^ΔL2 | ΔK278-Y293 | Reduced | Panel F | Fig. 7 |  |
| ^CI^ΔL3 | ΔI411-R436 | Normal | Panel F | Fig. 7 |  |
| ^CI^swapL3 | CI-I411-R436>GII-V410-R433 | Normal | Panel F | Fig. 7 |  |
| ^CI^ΔL4 | ΔE445-P461 | Normal | Panel F | Fig. 7 |  |
| ^CI^350_glyc_ | G350N | Normal | Panel F | Fig. 7 |  |
| ^CI^352_glyc_ | S352N | Undetectable |  | Fig. 7 |  |
| ^CI^463_glyc_ | W463N | Normal | Panel F | Fig. 7 |  |
| ^GII^SU | WT immunoadhesin, GII-K74 strain |  | Panel A | Fig. 3, 4, 5 |  |
| Kifu ^GII^SU | WT immunoadhesin produced in the presence of Kifunensine | Normal | Panel B | Fig. 3 |  |
| Kifu+Endo-H ^GII^SU | WT immunoadhesin produced in the presence of Kifunensine and treated with Endo-H | Normal | Panel B | Fig. 3 |  |
| ^GII^ΔN5 | N286>A | Reduced | Panel C | Fig. 3 |  |
| ^GII^ΔN6 | N311>A | Normal | Panel C | Fig. 3 |  |
| ^GII^ΔN7 | N346>A | Normal | Panel C | Fig. 3 |  |
| ^GII^ΔN7’ | N373>A | Reduced | Panel C | Fig. 3 |  |
| ^GII^ΔN9 | N404>A | Reduced | Panel C | Fig. 3 |  |
| ^GII^ΔN10 | N411>A | Reduced | Panel C | Fig. 3 |  |
| ^GII^ΔN9N10 | N404>A + N411>A | Insufficient | Panel C |  | Aggregates |
| ^GII^swap407 | 407RYNVNET413>KDIKSEI | Reduced | Panel D | Fig. 3 |  |
| ^GII^ΔRBDj | ΔF396-G480 | Normal | Panel E | Fig. 4 |  |
| ^GII^swapRBDj | GII-F396-G480>GI-F396-A482 | Reduced | Panel E | Fig. 7 |  |
| ^GII^ΔL2 | ΔK278-Y293 | Reduced | Panel F | Fig. 4 |  |
| ^GII^ΔL3 | ΔV410-R433 | Reduced | Panel F | Fig. 4 |  |
| ^GII^ΔL4 | ΔE442-P458 | Normal | Panel F | Fig. 4 |  |
| ^GII^263^glyc^ | D263N | Normal | Panel G | Fig. 4 |  |
| ^GII^426^glyc^ | H428>T | Normal | Panel I | Fig. 4 |  |
| ^GII^450^glyc^ | D450>N | Normal | Panel I | Fig. 4 |  |
| ^GII^A438-A443 | 438-REGKKE-443>AAGAAA | Insufficient |  |  |  |
| ^GII^459^glyc^ | E459>N | Normal | Panel I | Fig. 4 |  |
| ^GII^485^glyc^ | E485>N | Normal | Panel I | Fig. |  |
| ^GII^364^glyc^ | K364>N + G366>T | Reduced | Panel I |  | No nAb blocking and no cell binding |
| ^GII^351^glyc^ | L353T | Reduced | Panel H | Fig. 5, Supplementary Fig. 5 | Aggregates; experiments repeated with SEC purified immunoadhesin |
| ^GII^350^glyc^ | G350>N + K352>S | Reduced | Panel I | Fig. 5 | Aggregates, moderate |
| ^GII^349_+E_ | E inserted afterT348 | Reduced | Panel I | Fig. 5 |  |
| ^GII^swap333 | GII-L333-S345>CI-EQNERFLLNKLN | Reduced | Panel D | Fig. 5, Supplementary Fig. 6 |  |
| ^GII^swap345 | GII-S345-N351>GI-NNLTELTS | Normal | Panel D | Fig. 5, Supplementary Fig. 6 |  |
| ^GII^ΔT348-L353 | ΔT348-L353 + GG | Undetectable |  |  |  |
| ^GII^swap349 | GII-I349-N355>CI-SGTSVLKK | Insufficient |  |  |  |
| ^GII^E502A | E502>A | Reduced | Panel D | Fig. 5 |  |
| ^GII^L505N | L505>N | Reduced | Panel D | Fig. 5 |  |
| **Tagged proteins** | Proteins fused to a Strep-tag |  |  |  |  |
| ^MLV^SU | MLV SU, strain FB29 | Not applicable | A | Fig. 2 | Three bands are visible in non-reduced conditions that correspond to oligomers formed by the free cysteine thiol group [52]. |
| GII-K74 SU(m) | WT SU produced in mammalian cells | Not applicable | nd | Supplementary Fig. 2 | Purified by size exclusion chromatography [14] |
| GII-K74 SU (i) | WT SU produced in insect cells | Not applicable | nd | Supplementary Fig. 2 | Purified by size exclusion chromatography [14] |
| GII-K74 Ecto(i) | Env 91-907, produced in insect cells | Not applicable | nd | Supplementary Fig. 2 | Purified by size exclusion chromatography [14] |
| ^GII^K342A/R343A | K342>A+R343>A into GII-Ecto(i) | Normal | nd | Fig. 5 | Purified by size exclusion chromatography [14] |
| ^GII^R356A/R369A | R356>A+R369>A into GII-Ecto(i) | Normal | nd | Fig. 5 | Purified by size exclusion chromatography [14] |

^a^ aa positions indicated are those of each protein; for GII-K74, some differ from the CI-PFV based numbering (Supplementary Fig. 1). The symbols > and Δ designate aa substitutions and deletions, respectively.

^b^ The level of expression was assessed on crude supernatants of transfected cells and categorized as undetected, insufficient to perform the experiments, decreased, or normal relative to the WT counterpart.
