# Supplementary material for: Neutralization of zoonotic retroviruses by human antibodies: Genotype-specific epitopes within the receptor-binding domain from simian foamy virus
Source: PLoS Pathog. 2023 Apr 24;19(4):e1011339. doi: 10.1371/journal.ppat.1011339 (PMC10159361; doi:10.1371/journal.ppat.1011339)
Supplement: S2 Table — (DOCX) [file ppat.1011339.s002.docx]

## S2 Table. Plasma samples used for the neutralization study

| Participant | Ethnicity | SFV infection^a^ |
| --- | --- | --- |
| BAD448 | Bantu | GI |
| BAK132 | Pygmy | GI |
| LOBAK2 | Pygmy | GI |
| BAD551 | Bantu | GII |
| BAK133 | Pygmy | GII |
| BAK228 | Pygmy | GII |
| BAK232 | Pygmy | GII |
| MEBAK88 | Pygmy | GII |
| BAD348 | Bantu | GI+GII |
| BAD447 | Bantu | GI+GII |
| BAD468 | Bantu | GI+GII |
| BAK55 | Pygmy | GI+GII |

^a^ Participants were infected with a gorilla SFV of which the genotype was defined by PCR using primers located within SUvar [1]. Among the four individuals infected by both genotypes, only one (BAK55) was tested against both genotypes in epitope mapping experiments because his nAb titers were high against both genotypes; the three other samples were tested against a single viral genotype.

1. Lambert C, Couteaudier M, Gouzil J, Richard L, Montange T, Betsem E, et al. Potent neutralizing antibodies in humans infected with zoonotic simian foamy viruses target conserved epitopes located in the dimorphic domain of the surface envelope protein. PLoS Pathog. 2018;14:e1007293.
