# Supplementary material for: Neutralization of zoonotic retroviruses by human antibodies: Genotype-specific epitopes within the receptor-binding domain from simian foamy virus
Source: PLoS Pathog. 2023 Apr 24;19(4):e1011339. doi: 10.1371/journal.ppat.1011339 (PMC10159361; doi:10.1371/journal.ppat.1011339)
Supplement: S3 Table — (DOCX) [file ppat.1011339.s003.docx]

## S3 Table: Methods used to predict epitopic regions and to design the mutant SU proteins

| **Name** | **Prediction** | **Genotype-specific features** |
| --- | --- | --- |
| ^GII^ΔN5, ^GII^ΔN6, ^GII^ΔN7, ^GII^ΔN9 | Functional study [1] |  |
| ^GII^ΔN7’ | Presence varied according to the viral strain [2] | Absent from CI-PFV strain |
| ^GII^ΔN10, ^CI^ΔN10 | Functional study [1] | Genotype-specific localization of N10 [2] |
| ^GII^swap407 | Genotype-specific sequence before N10 | Genotype-specific localization of N10 [2] |
| ^GII^ΔRBDj, ^GII^swapRBDj, ^CI^ΔRBDj, ^CI^swapRBDj | Functional study [3] | ^GII^SU with ^CI^RBDj (^GII^swapRBDj) was expressed; ^CI^SU with ^GII^RBDj was not |
| ^GII^K342A/R343A,  ^GII^R356A/R369A | Functional study [4] | Mutations introduced in GII-K74 ectodomain; CI-PFV ectodomain is expressed at low levels |
| ^GII^ΔL2, ^CI^ΔL2 | Crystal structure [4] + disordered secondary structure^a^ |  |
| ^GII^ΔL3, ^CI^ΔL3, ^CI^L3swap, ^GII^ΔL4, ^CI^ΔL4 | Crystal structure ([4], cosubmitted) |  |
| ^GII^263^glyc^, ^GII^426^glyc^, ^GII^450^glyc^, ^GII^459^glyc^ | Genotype specific + CBtope^b^ |  |
| ^GII^351^glyc^, ^GII^364^glyc^, ^GII^485^glyc^ | Genotype specific + CBtope^b^ + disordered secondary structure^a^ | Loop size around aa351 differs between genotypes |
| ^GII^350^glyc^, ^GII^349_+E_, ^GII^swap345, ^GII^swap333, ^GII^E502A, ^GII^L505N, ^CI^G350^glyc^ | Designed after testing ^GII^351^glyc^ |  |
| ^CI^463^glyc^ | Designed after testing GII ^GII^459^glyc^ |  |

^a^ The Protein Homology/analogY Recognition Engine V 2.0 (Phyre2) web portal was used for secondary structure prediction [5]. Disordered secondary structures predicted by the software were considered to define epitopic regions to be tested.

^b^ CBtope software predicts conformational B-cell epitopes on the basis of their primary sequence (<http://crdd.osdd.net/raghava/cbtope/>, [6]). We applied the recommended parameters, i.e. 19 aa-long window, -0.3 threshold, and considered values ≥ 4 as potential epitopes

1. Luftenegger D, Picard-Maureau M, Stanke N, Rethwilm A, Lindemann D. Analysis and function of prototype foamy virus envelope N glycosylation. J Virol. 2005;79:7664-72.

2. Richard L, Rua R, Betsem E, Mouinga-Ondeme A, Kazanji M, Leroy E, et al. Cocirculation of two *env* molecular variants, of possible recombinant origin, in gorilla and chimpanzee simian foamy virus strains from Central Africa. J Virol. 2015;89:12480-91.

3. Duda A, Luftenegger D, Pietschmann T, Lindemann D. Characterization of the prototype foamy virus envelope glycoprotein receptor-binding domain. J Virol. 2006;80:8158-67.

4. Fernandez I, Dynesen LT, Coquin Y, Pederzoli R, Brun D, Haouz A, et al. The crystal structure of a simian Foamy Virus receptor binding domain provides clues on the entry to host cells. Nat Comm. 2023;14:1262.

5. Kelley LA, Mezulis S, Yates CM, Wass MN, Sternberg MJ. The Phyre2 web portal for protein modeling, prediction and analysis. Nat Protoc. 2015;10:845-58.

6. Ansari HR, Raghava GP. Identification of conformational B-cell Epitopes in an antigen from its primary sequence. Immunome Res. 2010;6:6.
