# Supplementary material for: Neutralization of zoonotic retroviruses by human antibodies: Genotype-specific epitopes within the receptor-binding domain from simian foamy virus
Source: PLoS Pathog. 2023 Apr 24;19(4):e1011339. doi: 10.1371/journal.ppat.1011339 (PMC10159361; doi:10.1371/journal.ppat.1011339)
Supplement: S4 Table — (DOCX) [file ppat.1011339.s004.docx]

## S4 Table. Plasma samples used for the ELISA assays

| Participant | Ethnicity | SFV infection^a^ | Fig. 8^b^ | Supplementary Fig. 7^b^ |
| --- | --- | --- | --- | --- |
| BAD356 | Bantu | Uninfected |  | X |
| BAK141 | Pygmy | Uninfected |  | X |
| BAK183 | Pygmy | Uninfected |  | X |
| BAK279 | Pygmy | Uninfected |  | X |
| MEBAK195 | Pygmy | GI | X |  |
| BAD448 | Bantu | GI | X | X |
| BAD463 | Bantu | GI | X |  |
| BAK132 | Pygmy | GI | X | X |
| BAK56 | Pygmy | GI | X |  |
| BAK82 | Pygmy | GI | X |  |
| LOBAK2 | Pygmy | GI | X | X |
| BAD551 | Bantu | GII | X | X |
| BAK133 | Pygmy | GII |  | X |
| BAK232 | Pygmy | GII | X | X |
| MEBAK88 | Pygmy | GII |  | X |
| BAD348 | Bantu | GI+GII | X |  |
| BAD447 | Bantu | GI+GII | X |  |
| BAD468 | Bantu | GI+GII | X | X |
| BOBAK153 | Pygmy | GI+GII | X |  |
| BAD456 | Bantu | GI+GII | X |  |
| BAK177 | Pygmy | GI+GII | X |  |
| BAK55 | Pygmy | GI+GII | X |  |
| BAK74 | Pygmy | GI+GII | X |  |

^a^ Participants were infected with a gorilla SFV of which the genotype (GI or GII) was defined by PCR using primers located within SUvar [1]. Some participants were coinfected by strains from both genotypes (GI+GII). ^b^ The samples used are indicated for each of the two sets of peptides tested and presented in Fig.8 and supplementary Fig. 7.

1. Lambert C, Couteaudier M, Gouzil J, Richard L, Montange T, Betsem E, et al. Potent neutralizing antibodies in humans infected with zoonotic simian foamy viruses target conserved epitopes located in the dimorphic domain of the surface envelope protein. PLoS Pathog. 2018;14:e1007293.
