# Supplementary material for: Neutralization of zoonotic retroviruses by human antibodies: Genotype-specific epitopes within the receptor-binding domain from simian foamy virus
Source: PLoS Pathog. 2023 Apr 24;19(4):e1011339. doi: 10.1371/journal.ppat.1011339 (PMC10159361; doi:10.1371/journal.ppat.1011339)
Supplement: S5 Table — (DOCX) [file ppat.1011339.s005.docx]

## S5 Table. Synthetic peptides used to search for linear epitopes

| Peptide name | Position^a^ | Sequence^b^ | Length | Prediction^c^ |
| --- | --- | --- | --- | --- |
| BAD468-247 | 247-270 | _RR_PSEELIADQCPLPGYHAGVEYTTQ_RR_ | 28 | Parker |
| BAD468-267 | 267-284 | RYTTQAIWDYYIKVEITRP | 19 | Genotype-specific |
| BAD468-280 | 280-300 | EITRPKNWTSYAQYGNARLGS_R_ | 22 | Parker + Lbtope |
| BAD468-308 | 308-337 | RKNFTHVLFCSDQLYAKWYNIENTLLKNEE_R_ | 31 | Ellipro |
| BAD468-329 | 329-342 | _R_ENTLLKNEELLQKK | 15 | Parker |
| Bad468-340 | 340-357 | LQKKLNNLTELTSLLKKR | 18 | Genotype-specific |
| BAD468-350 | 350-374 | TSLLKKRALPRTWTTQGKNNLFRNI | 25 | Lbtope |
| BAD468-399 | 399-418 | RWEGDCNYTKDKISEIVPQCKR | 22 | Parker |
| BAD468-411 | 411-432 | RSEIVPQCKGFYNNSKWMHMHPYR | 24 | Parker |
| BAD468-425 | 424-444 | SKWMHMHPYACRFWRNKNEKE | 21 | Lbtope |
| BAD468-435 | 435-454 | RFWRNKNEKEETKCDGRDDN | 20 | Lbtope |
| BAD468-441 | 441-460 | RNEKEETKCDGRDDNKCLYYPRR | 23 | Parker + Lbtope |
| BAD468-450 | 450-472 | RRGRDDNKCLYYPLWDSPEATYDFGRRR | 28 | Parker + Lbtope |
| BAD468-487 | 487-512 | SSKQIRQQDYEVYSIYQECKLASRIH | 26 | Lbtope |
| BAK74-247 | 247-270 | RRPNEGLIADQCPLPGLADVSFYPYQRRR | 29 | Parker |
| BAK74-267 | 267-284 | RYPYQAIWDYYAKIENIRP | 19 | Genotype-specific |
| BAK74-280 | 280-300 | RENIRPANWTSSKLYGKARMGSR | 23 | Parker + Lbtope |
| BAK74-308 | 308-338 | RNINNTHILFCSDVLYSKWYNLQNSILQNENR | 32 | Ellipro |
| BAK74-330 | 330-348 | RQNSILQNENELTKRLSNLT | 20 | Parker |
| Bak74-339 | 339-355 | ELTKRLSNLTIGNKLKN | 17 | Genotype-specific |
| BAK74-350 | 350-374 | GNKLKNRALPYEWAKGGLNRLFRNI | 25 | Lbtope |
| BAK74-399 | 399-418 | RWEGDCNITRYNVNETVPECKR | 22 | Parker |
| BAK74-411 | 411-430 | RNETVPECKDFPHRRFNDHPYR | 22 | Lbtope |
| BAK74-425 | 424-442 | RRFNDHPYSCRLWRYREGKE | 20 | Lbtope |
| BAK74-435 | 433-452 | RLWRYREGKEEVKCLTSDHTR | 21 | Lbtope |
| BAK74-441 | 439-458 | REGKEEVKCLTSDHTRCLYYPRR | 23 | Parker + Lbtope |
| BAK74-449 | 449-470 | RRSDHTRCLYYPEYSNPEALFDFGRR | 26 | Parker + Lbtope |
| BAK74-485 | 485-510 | RESTSIRQQDYEVYSIYQECKLASKTYR | 28 | Lbtope |
| PFV-37 | 251-265 | LIADQCPLPGYHAGL | 15 | Genotype-specific |
| PFV-41 | 271-285 | SIWDYYIKVESIRPA | 15 | Genotype-specific |
| PFV-46 | 296-310 | ARLGSFYIPSSLRQI | 15 | Genotype-specific |
| PFV-55 | 341-355 | LNKLNNLTSGTSVLK | 15 | Genotype-specific |
| PFV-65 | 391-405 | NTSYYSFSLWEGDCN | 15 | Genotype-specific |
| PFV-66 | 396-410 | SFSLWEGDCNFTKDM | 15 | Genotype-specific |
| PFV-80 | 466-470 | PESTYDFGYLAYQKN | 15 | Genotype-specific |
| PFV-81 | 471-485 | DFGYLAYQKNFPSPI | 15 | Genotype-specific |
| PFV-82 | 476-490 | AYQKNFPSPICIEQQ | 15 | Genotype-specific |
| L1-GI | 258-269 | _R_LPGYHAGVEYTT_R_ | 14 | RBD structure |
| L1-GII | 258-269 | _R_LPGLADVSFYPY_R_ | 14 | RBD structure |
| L2-GI | 279-288 | VEITRPKNWT_R_ | 11 | RBD structure |
| L2-GII | 279-288 | IENIRPANWT_R_ | 11 | RBD structure |
| L3-GI | 411-435 | SEIVPQCKGFYNNSKWMHMHPYACR | 25 | RBD structure |
| L3-GII | 410-433 | VNETVPECKDFPHRRFNDHPYSCR | 24 | RBD structure |
| L4-GI | 447-457 | _R_KCDGRDDNKCL | 12 | RBD structure |
| L4-GII | 445-455 | _R_KCLTSDHTRCL | 12 | RBD structure |

^a^ Positions refer to each viral sequence.

^b^ Subscript characters indicate residues added to increase peptide solubility.

^c^ Linear B-cell epitopes were predicted using the software available on the Immune Epitope Data Base (<http://tools.iedb.org/bcell/>): LBtope ([1] and Parker hydrophilicity prediction replaced by the Bepipred program [2] and Ellipro [3]). Genotype-specific sequences were manually defined. After resolution of the RBD structure [4], eight novel peptides overlapping the four loops were synthesized.

1. Singh H, Ansari HR, Raghava GP. Improved method for linear B-cell epitope prediction using antigen's primary sequence. PLoS One. 2013;8:e62216.

2. Larsen JE, Lund O, Nielsen M. Improved method for predicting linear B-cell epitopes. Immunome Res. 2006;2:2.

3. Ponomarenko J, Bui HH, Li W, Fusseder N, Bourne PE, Sette A, et al. ElliPro: a new structure-based tool for the prediction of antibody epitopes. BMC Bioinformatics. 2008;9:514.

4. Fernandez I, Dynesen LT, Coquin Y, Pederzoli R, Brun D, Haouz A, et al. The crystal structure of a simian Foamy Virus receptor binding domain provides clues on the entry to host cells. Nat Comm. 2023;14:1262.
