# Supplementary material for: Neutralization of zoonotic retroviruses by human antibodies: Genotype-specific epitopes within the receptor-binding domain from simian foamy virus
Source: PLoS Pathog. 2023 Apr 24;19(4):e1011339. doi: 10.1371/journal.ppat.1011339 (PMC10159361; doi:10.1371/journal.ppat.1011339)
Supplement: S1 Fig — Env sequences from CI-PFV, GI-D468, and GII-K74 strains were aligned using CLC Mainworkbench software. Identical residues are indicated with dots. Boundaries of the leader peptide (LP), surface protein (SU), transmembrane protein (TM), receptor binding domain (RBD)1, RBDj, and RBD2 are indicated over the sequences. The RBDj domain is highlighted by italic characters and the SUvar domain is highlighted by the grey colored background. (DOCX) [file ppat.1011339.s006.docx]

## S1 Fig. CI-PFV, GI-D468, and GII-K74 Env sequence alignment

|  | **\|LP** |  |
| --- | --- | --- |
| CI-PFV | MAPPMTLQQW IIWKKMNKAH EALQNTTTVT EQQKEQIILD IQNEEVQPTR | 50 |
| GI-D468 | .....S.... ...N..HQ.. Q....S.L.. .E.......E ....D.V... | 50 |
| GII-K74 | .....S.... ...N..HQ.. Q....S.L.. .E.......E ....D.I... | 50 |
|  |  |  |
| CI-PFV | RDKFRYLLYT CCATSSRVLA WMFLVCILLI IVLVSCFVTI SRIQWNKDIQ | 100 |
| GI-D468 | M.RVK.F... .......... ..L.A...F. .II....I.L .......... | 100 |
| GII-K74 | M.RVK.F... .......... ..L.A...F. .II....I.L .......... | 100 |
|  | **\|SU** |  |
| CI-PFV | VLGPVIDWNV TQRAVYQPLQ TRRIARSLRM QHPVPKYVEV NMTSIPQGVY | 150 |
| GI-D468 | .......... .......... L.....A..A .......... .........F | 150 |
| GII-K74 | .......... .......... L.....A..A .......... .........F | 150 |
|  |  |  |
| CI-PFV | YEPHPEPIVV KERVLGLSQI LMINSENIAN NANLTQEVKK LLTEMVNEEM | 200 |
| GI-D468 | .Q......IH T........V .......V.. S...S..T.A .....I.... | 200 |
| GII-K74 | .Q......IH T........V .......V.. S...S..T.A .......... | 200 |
|  | **╟RBD1** \|SUvar |  |
| CI-PFV | QSLSDVMIDF EIPLGDPRDQ EQYIHRKCYQ EFANCYLVKY KEPKPWPKEG | 250 |
| GI-D468 | .......... .......... .......... ...H...... .T.Q...S.E | 250 |
| GII-K74 | .G........ .......... .......... ...H...... .T.Q...N.. | 250 |
|  |  |  |
| CI-PFV | LIADQCPLPG YHAGLTYNRQ SIWDYYIKVE SIRPANWTTK SKYGQARLGS | 300 |
| GI-D468 | .......... ....VE.TT. A......... IT..K...SY AQ..N..... | 300 |
| GII-K74 | .......... LADVSF.PY. A.....A.I. N.......SS KL..K..M.. | 300 |
|  |  |  |
| CI-PFV | FYIPSSLRQI NVSHVLFCSD QLYSKWYNIE NTIEQNERFL LNKLNNLTSG | 350 |
| GI-D468 | .F..PHV.K- .FT....... ...A...... ..LLK..EL. QK......EL | 349 |
| GII-K74 | Y...KR..N. .NT.I..... V.......LQ .S.L...NE. TKR.S...-I | 349 |
|  | **╟***RBDj* |  |
| CI-PFV | TSVLKKRALP KDWSSQGKNA LFREINVLDI CSKPESVILL NTSYYS*FSLW* | 400 |
| GI-D468 | ..L....... RT.TT....N ...N.T...V .NR..M.L.. .I..DL.... | 399 |
| GII-K74 | GNK..N.... YE.AKG.L.R ...N.S...V ..R..M.L.. .KT..T.... | 399 |
|  |  |  |
| CI-PFV | *EGDCNFTKDM ISQLVPECDG FYNNSKWMHM HPYACRFWRS KNEKEETKCR* | 450 |
| GI-D468 | *.....Y...K ..EI..Q.K. .......... .........N .........D* | 449 |
| GII-K74 | *.....I.RYN VNET....KD .PHRR--FND ...S..L..Y REG...V..L* | 447 |
|  | **╟RBD2** \| |  |
| CI-PFV | *DGETKRCLYY PLWDSPESTY DFGYLAYQKN FPS*PICIEQQ KIRDQDYEVY | 500 |
| GI-D468 | *GRDDNK.... .......A.. ...F....N. ..A*....SSK Q..Q...... | 499 |
| GII-K74 | *TSDHT..... .EYSN..ALF ...F.S.MR. ..G*.Q...ST S..Q...... | 497 |
|  |  |  |
| CI-PFV | SLYQERKIAS KAYGIDTVLF SLKNFLNYTG TPVNEMPNAR AFVGLIDPKF | 550 |
| GI-D468 | .I...C.L.. RIH...S... .......... K......... .......... | 549 |
| GII-K74 | .I...C.L.. .T....S... .......... K......... .......... | 547 |
|  | ╢ **\|TM** |  |
| CI-PFV | PPSYPNVTRE HYTSCN—-NR KRRSVDNNYA KLRSMGYALT GAVQTLSQIS | 598 |
| GI-D468 | ..T...I..D Q.QG..INQ. RK.E.N...S .......... ......A... | 599 |
| GII-K74 | ..T...I..D Q.QG..INQ. RK.E.N...S .......... ......A... | 597 |
|  |  |  |
| CI-PFV | DINDENLQQG IYLLRDHVIT LMEATLHDIS VMEGMFAVQH LHTHLNHLKT | 648 |
| GI-D468 | ....Q..... .......IV. .......... I......... V.......R. | 649 |
| GII-K74 | ....Q..... .......IV. .......... I......... V.......R. | 647 |
|  |  |  |
| CI-PFV | MLLERRIDWT YMSSTWLQQ QLQKSDDEMKV IKRIARSLVY YVKQTHSSPT | 698 |
| GI-D468 | ..M....... ....S...T ........... ...T...... .....YN.L. | 699 |
| GII-K74 | ..M....... ....S...T ........... ...T...... .....YN.L. | 697 |
|  |  |  |
| CI-PFV | ATAWEIGLYY ELVIPKHIY LNNWNVVNIGH LVKSAGQLTH VTIAHPYEII | 748 |
| GI-D468 | .......... ..I..R... ....Q...... .I........ ..LS...... | 749 |
| GII-K74 | .......... ..I..R... ....QI..... .I........ ..LS...... | 747 |
|  |  |  |
| CI-PFV | NKECVETIYL HLEDCTRQDY VICDVVKIVQ PCGNSSDTSD CPVWAEAVKE | 798 |
| GI-D468 | .R..SN.L.. ...E.R.L.. .......... .......S.. ......P... | 799 |
| GII-K74 | .R..SN.L.. ...E.R.L.. .......... .......S.. ......P... | 797 |
|  |  |  |
| CI-PFV | PFVQVNPLKN GSYLVLASST DCQIPPYVPS IVTVNETTSC FGLDFKRPLV | 848 |
| GI-D468 | .H..IS.... .......... .......... V.......Q. ..VT..K... | 849 |
| GII-K74 | .H..IS.... .......... .......... V.......Q. ..VT..K... | 847 |
|  |  |  |
| CI-PFV | AEERLSFEPR LPNLQLRLPH LVGIIAKIKG IKIEVTSSGE SIKEQIERAK | 898 |
| GI-D468 | ...KT.L..Q ..H....... .......... .......... ...D.L.... | 899 |
| GII-K74 | ...KT.L..Q ..H....... .......... .......... ...D.L.... | 897 |
|  |  |  |
| CI-PFV | AELLRLDIHE GDTPAWIQQL AAATKDVWPA AASALQGIGN FLSGTAQGIF | 948 |
| GI-D468 | .......... .......... ....E..... .....K.... ..T.A...L. | 949 |
| GII-K74 | .......... .......R.. ....E..... .....K.... ..T.A...L. | 947 |
|  |  |  |
| CI-PFV | GTAFSLLGYL KPILIGVGVI LLVILIFKIV SWIPTKKKNQ | 988 |
| GI-D468 | .....I.... ......I.I. I........L K...I.R.S. | 989 |
| GII-K74 | .....I.... ......I.I. I........L K...I...S. | 987 |

Env sequences from CI-PFV, GI-D468, and GII-K74 strains were aligned using CLC Mainworkbench software. Identical residues are indicated with dots. Boundaries of the leader peptide (LP), surface protein (SU), transmembrane protein (TM), receptor binding domain (RBD)1, RBDj, and RBD2 are indicated over the sequences. The RBDj domain is highlighted by italic characters and the SUvar domain is highlighted by the grey colored background.
