# Supplementary material for: Neutralization of zoonotic retroviruses by human antibodies: Genotype-specific epitopes within the receptor-binding domain from simian foamy virus
Source: PLoS Pathog. 2023 Apr 24;19(4):e1011339. doi: 10.1371/journal.ppat.1011339 (PMC10159361; doi:10.1371/journal.ppat.1011339)
Supplement: S2 Fig — A. Schematic representation of SFV Env constructs tested for expression in mammalian and/or insect cells. SFV Env is shown in grey; the RBD is shown in dark grey. The dark green segment represents the Twin-Strep-tag and the light green segment the murine Fc domain. The outcomes of expression assays for CI-PFV, GI-D468, and GII-K74 Env-derived proteins are summarized in the table. CI-PFV immunoadhesin (CISU) was the only well-expressed genotype I Env protein and we therefore used immunoadhesins for the project. B. GII-specific plasma samples from four individuals were diluted to their ≈ IC90 and incubated with Env-derived proteins at concentrations ranging from 200 to 0.02 nM. The mix was then added to FVVs expressing GII-K74 Env before titration. The relative infectivity is presented as a function of the protein concentration. Production in mammalian or insect cells is indicated in the legend with (m) and (i) suffixes, respectively. The oligomerisation/chimeric state is indicated in parentheses. The left and right panels present independent experiments. The inhibition of anti-GII nAbs by SU was independent of the nature of the producing cell (GIISU[mono]m vs. GIISU[mono]i, left panels), dimerization through fusion with the immunoglobulin constant domain (GIISU[mono]m vs. GIISU[Ig]m, left panels), and trimerization when expressed as an ectodomain in insect cells (GIISU[mono]i vs. GIIEcto[trimer]i, right panels). Genotype-mismatched immunoadhesins (CISU[Ig]m) failed to inhibit the nAbs (blue curves, left panels). One representative experiment is shown for four individuals; experiments were carried out in triplicate, means and standard error to the mean are presented on the graphs. (DOCX) [file ppat.1011339.s007.docx]

## S2 Fig. Recombinant SFV Env oligomerization and mammalian-specific glycolysation do not affect the capacity to inhibit GII-specific nAbs

A. Schematic representation of SFV Env constructs tested for expression in mammalian and/or insect cells. SFV Env is shown in grey; the RBD is shwon in dark grey. The dark green segment represents the Twin-Strep-tag and the light green segment the murine Fc domain. The outcomes of expression assays for CI-PFV, GI-D468, and GII-K74 Env-derived proteins are summarized in the table. CI-PFV immunoadhesin (^CI^SU) was the only well-expressed genotype I Env protein and we therefore used immunoadhesins for the project. B. GII-specific plasma samples from four individuals were diluted to their ≈ IC_90_ and incubated with Env-derived proteins at concentrations ranging from 200 to 0.02 nM. The mix was then added to FVVs expressing GII-K74 Env before titration. The relative infectivity is presented as a function of the protein concentration. Production in mammalian or insect cells is indicated in the legend with (m) and (i) suffixes, respectively. The oligomerisation/chimeric state is indicated in parentheses. The left and right panels present independent experiments. The inhibition of anti-GII nAbs by SU was independent of the nature of the producing cell (^GII^SU[mono]^m^ vs. ^GII^SU[mono]^i^, left panels), dimerization through fusion with the immunoglobulin constant domain (^GII^SU[mono]^m^ vs. ^GII^SU[Ig]^m^, left panels), and trimerization when expressed as an ectodomain in insect cells (^GII^SU[mono]^i^ vs. ^GII^Ecto[trimer]^i^_,_ right panels). Genotype-mismatched immunoadhesins (^CI^SU[Ig]^m^) failed to inhibit the nAbs (blue curves, left panels).
