# Supplementary material for: Neutralization of zoonotic retroviruses by human antibodies: Genotype-specific epitopes within the receptor-binding domain from simian foamy virus
Source: PLoS Pathog. 2023 Apr 24;19(4):e1011339. doi: 10.1371/journal.ppat.1011339 (PMC10159361; doi:10.1371/journal.ppat.1011339)
Supplement: S3 Fig — Western-blotting analysis of WT SU protein and immunoadhesins. Mammalian cell supernatants collected 72 h post-transfection were heat-denaturated before immunoblotting with either anti-Strep-tag antibody (A and C) or an anti-SU antibody (B). GIISU was expressed as an immunoadhesin without a Strep-tag, an immunoadhesin with a Strep-tag, as monomeric SU with a Strep-tag;s CISU was expressed as an immunoadhesin with a Strep-tag (A and B). MLVSU was expressed as SU fused to a Strep-tag (C). For the CI and GII SUs, two bands are visible, in accordance with the results of other reports [27]. (DOCX) [file ppat.1011339.s008.docx]

## S3 Fig. Western-blot analysis of WT SU proteins used in the study

Western-blotting analysis of WT SU protein and immunoadhesins. Mammalian cell supernatants collected 72 h post-transfection were heat-denaturated before immunoblotting with either anti-Strep-tag antibody (A and C) or an anti-SU antibody (B). ^GII^SU was expressed as an immunoadhesin without a Strep-tag, an immunoadhesin with a Strep-tag, as monomeric SU with a Strep-tag;s ^CI^SU was expressed as an immunoadhesin with a Strep-tag (A and B). ^MLV^SU was expressed as SU fused to a Strep-tag (C). For the CI and GII SUs, two bands are visible, in accordance with the results of other reports [1].

1. Herchenroder O, Moosmayer D, Bock M, Pietschmann T, Rethwilm A, Bieniasz PD, et al. Specific binding of recombinant foamy virus envelope protein to host cells correlates with susceptibility to infection. Virology. 1999;255:228-36.
