# Supplementary material for: Neutralization of zoonotic retroviruses by human antibodies: Genotype-specific epitopes within the receptor-binding domain from simian foamy virus
Source: PLoS Pathog. 2023 Apr 24;19(4):e1011339. doi: 10.1371/journal.ppat.1011339 (PMC10159361; doi:10.1371/journal.ppat.1011339)
Supplement: S4 Fig — To verify protein purity and aggregate formation, 1.5 μg of purified proteins were heat-denaturated at 70°C for 10 min, with or without DTT. Samples were loaded onto a precast NuPAGE 4–12% Bis-Tris gel and the proteins separated by electrophoresis. Gels were then stained with Coomassie blue and imaged using a G:BOX (Syngene). A western-blot control was performed for all affinity-purified proteins. The constructs are listed in S1 Table and their names are indicated over the images. (DOCX) [file ppat.1011339.s009.docx]

## S4 Fig. Purity of proteins used in the study assessed by Coomassie blue gel staining


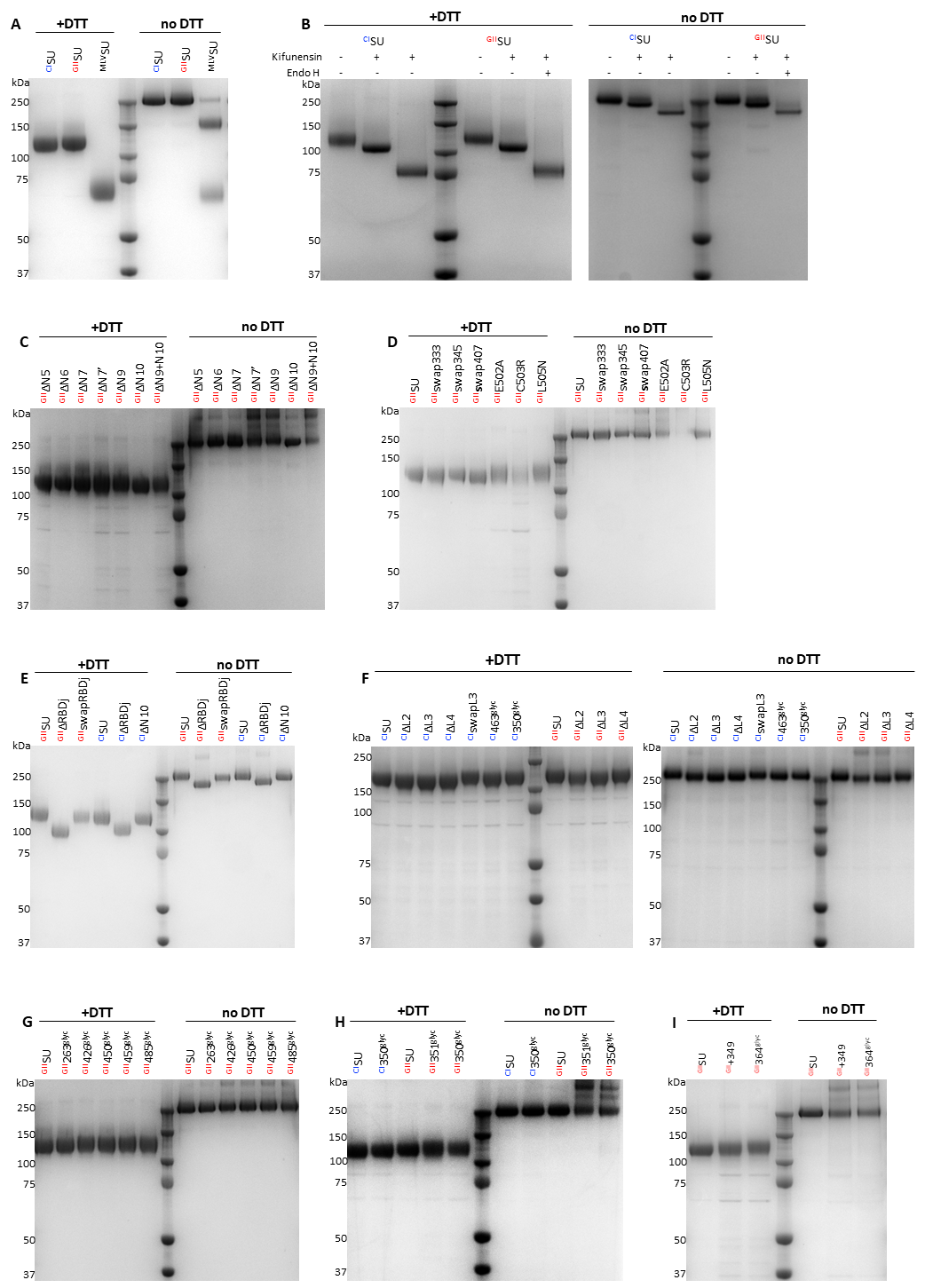


To verify protein purity and aggregate formation, 1.5 µg of purified proteins were heat-denaturated at 70°C for 10 min, with or without DTT. Samples were loaded onto a precast NuPAGE 4-12% Bis-Tris gel and the proteins separated by electrophoresis. Gels were then stained with Coomassie blue and imaged using a G:BOX (Syngene). A western-blot control was performed for all affinity-purified proteins. The constructs are listed in supplementary Table 1 and their names are indicated over the images.
