# Supplementary material for: Neutralization of zoonotic retroviruses by human antibodies: Genotype-specific epitopes within the receptor-binding domain from simian foamy virus
Source: PLoS Pathog. 2023 Apr 24;19(4):e1011339. doi: 10.1371/journal.ppat.1011339 (PMC10159361; doi:10.1371/journal.ppat.1011339)
Supplement: S5 Fig — 293-F cells were transfected with the plasmid encoding GII351glyc. The supernatant was collected after 72 h of culture and the SU was affinity purified using the Strep-Tag fused to the C-terminus. Then, half the volume was purified by size exclusion chromatography. A. The affinity-purified and four fractions of chromatography-purified GII351glyc were analyzed on a Coomassie-stained gel, with or without reducing treatment. High molecular weight proteins were present in the affinity purified sample and the first two SEC fractions. B to E. The plasma samples from four individuals infected with a GII SFV were diluted to their ≈ IC90 and incubated with SU at concentrations ranging from 60 to 0.02 nM. The mix was then added to FVVs expressing the GII Env before titrating infectivity. The relative infectivity is presented as a function of SU concentration. The addition of GIISU (black symbols) inhibited the action of the nAbs, whereas affinity-purified GII351glyc did not (green closed symbols). To exclude that GII351glyc aggregation led to epitope masking, the chromatography-purified fractions 3 and 4 (GII351glyc [SEC], green open symbols) were pooled, concentrated, and tested in parallel. These contained no aggregates (panel A) but were unable to block nAbs from the four individuals. One representative experiment is shown for four individuals; experiments were carried out in triplicate, means and standard error to the mean are presented on the graphs. (DOCX) [file ppat.1011339.s010.docx]

## S5 Fig. The ^GII^351_glyc_ SU is unable to block nAbs – exclusion of a nonspecific effect of protein aggregation

293-F cells were transfected with the plasmid encoding ^GII^351_glyc_. The supernatant was collected after 72 h of culture and the SU was affinity purified using the Strep-Tag fused to the C-terminus. Then, half the volume was purified by size exclusion chromatography. A. The affinity-purified and four fractions of chromatography-purified ^GII^351_glyc_ were analyzed on a Coomassie-stained gel, with or without reducing treatment. High molecular weight proteins were present in the affinity purified sample and the first two SEC fractions. B to E. The plasma samples from four individuals infected with a GII SFV were diluted to their ≈ IC_90_ and incubated with SU at concentrations ranging from 60 to 0.02 nM. The mix was then added to FVVs expressing the GII Env before titrating infectivity. The relative infectivity is presented as a function of SU concentration. The addition of ^GII^SU (black symbols) inhibited the action of the nAbs, whereas affinity-purified ^GII^351_glyc_ did not (green closed symbols). To exclude that ^GII^351_glyc_ aggregation led to epitope masking, the chromatography-purified fractions 3 and 4 (^GII^351_glyc_ [SEC], green open symbols) were pooled, concentrated, and tested in parallel. These contained no aggregates (panel A) but were unable to block nAbs from the four individuals.
