# Supplementary material for: Neutralization of zoonotic retroviruses by human antibodies: Genotype-specific epitopes within the receptor-binding domain from simian foamy virus
Source: PLoS Pathog. 2023 Apr 24;19(4):e1011339. doi: 10.1371/journal.ppat.1011339 (PMC10159361; doi:10.1371/journal.ppat.1011339)
Supplement: S6 Fig — GIISU in which residues 333–345 or 345–351 were replaced by those from the GI-D468 strain were tested for their capacity to block nAbs from GI-specific plasma samples. A. GIIswap333; B. GIIswap345 C. All mutants were tested against four plasma samples. For each plasma sample, two parameters were calculated as descibed in material and methods and displayed on the graphs. IC50 is presented as a function of MaxI for the CISU and mutant SU. The IC50 and MaxI values of CISU are presented as open symbols and are those from the same experiment in which the mutant SUs were tested. For mutant SUs, the symbols are colored according to the IC50 and MaxI thresholds used to statistically define significant differences from CISU. (DOCX) [file ppat.1011339.s011.docx]

## S6 Fig. The GII chimeric SUs in which aa 333-345 or 345-351 were replaced by GI sequences are unable to block GI-specific nAbs

^GII^SU in which residues 333-345 or 345-351 were replaced by those from the GI-D468 strain were tested for their capacity to block nAbs from GI-specific plasma samples. A. ^GII^swap333; B. ^GII^swap345 C. All mutants were tested against four plasma samples. For each plasma sample, the IC_50_ is presented as a function of MaxI for the ^CI^SU and mutant SU. The IC_50_ and MaxI values of ^CI^SU are presented as open symbols and are those from the same experiment in which the mutant SUs were tested. For mutant SUs, the symbols are colored according to the IC_50_ and MaxI thresholds used to statistically define significant differences from ^CI^SU.
