# Supplementary material for: Neutralization of zoonotic retroviruses by human antibodies: Genotype-specific epitopes within the receptor-binding domain from simian foamy virus
Source: PLoS Pathog. 2023 Apr 24;19(4):e1011339. doi: 10.1371/journal.ppat.1011339 (PMC10159361; doi:10.1371/journal.ppat.1011339)
Supplement: S7 Fig — Seventeen plasma samples from African hunters were tested for binding to 37 peptides located in the SUvar domain (S5 Table). The summary graph shows positive responses (ΔOD, y-axis) plotted against peptides identified by the position of their first aa (x-axis). Plasma samples are identified by a color code corresponding to the genotype(s) of the infecting strains (blue: infected with a GI strain, red: infected with a GII strain, purple: infected by strains of both genotypes [7]). Left, peptides spanning GI SUvar; right, peptides spanning GII SUvar; the RBDj region is indicated by the lighter color. Detailed binding activity is shown on the aligned SU sequences; the RBDj sequence is highlighted in italic characters; the sequence covered by peptides is highlighted by grey background. Recognized peptides are underscored and designated by the same letters as those used in panel A. Reactive plasma samples are indicated above the sequence and colored according to the genotype(s) of the infecting strains. Six plasma samples from SFV-infected individuals reacted against seven peptides located in the RBDj region (BAD551, BAK55, BAK56, BAK74, BAK82, and BAK132) and two samples reacted against a peptide located in the RBD1 or RBD2 subdomains (BAD468 and BAK56, respectively). Plasma antibody binding to the peptides was not genotype-specific. For example, sample BAK56 reacted against peptide b (399W-K418) from the GI-D468 and GII-K74 strains. (DOCX) [file ppat.1011339.s012.docx]

## S7 Fig. Only a small proportion of plasma samples bind to peptides covering the SUvar domain

| **CI-PFV** LVKYKEPKPW PKEGLIADQC PLPGYHAGLT YNRQSIWDYY | 276 |
| --- | --- |
| BAD468[g1g2] |  |
| **GI-D468** LVKYKTPQPW PSEELIADQC PLPGYHAGVE YTTQAIWDYY | 276 |
|  |  |
| **GII-K74** LVKYKTPQPW PNEGLIADQC PLPGLADVSF YPYQAIWDYY | 276 |
|  |  |
| IKVESIRPAN WTTKSKYGQA RLGSFYIPSS LRQINVSHVL FCSDQLYSKW | 326 |
| IKVEITRPKN WTSYAQYGNA RLGSFFIPPH VRK-NFTHVL FCSDQLYAKW | 325 |
| AKIENIRPAN WTSSKLYGKA RMGSYYIPKR LRNINNTHIL FCSDVLYSKW | 326 |
|  |  |
| YNIENTIEQN ERFLLNKLNN LTSGTSVLKK RALPKDWSSQ GKNALFREIN | 376 |
| YNIENTLLKN EELLQKKLNN LTELTSLLKK RALPRTWTTQ GKNNLFRNIT | 375 |
| YNLQNSILQN ENELTKRLSN LT-IGNKLKN RALPYEWAKG GLNRLFRNIS | 375 |
|  |  |
| VLDICSKPES VILLNTSYYS *FSLWEGDCNF TKDMISQLVP ECDGFYNNSK* | 426 |
| b-BAK56[g1] |  |
| VLDVCNRPEM VLLLNISYDL *FSLWEGDCNY TKDKISEIVP QCKGFYNNSK* | 425 |
| b-BAK56[g1] BAK82[g1] |  |
| VLDVCSRPEM VLLLNKTYYT *FSLWEGDCNI TRYNVNETVP ECKDFPHRR-* | 424 |
|  |  |
| *WMHMHPYACR FWRSKNEKEE TKCRDGETKR CLYYPLWDSP ESTYDFGYLA* | 476 |
| c-BAD551[g2] BAK55[g1g2] BAK74[g1g2] |  |
| *WMHMHPYACR FWRNKNEKEE TKCDGRDDNK CLYYPLWDSP EATYDFGFLA* | 475 |
| *-FNDHPYSCR LWRYREGKEE VKCLTSDHTR CLYYPEYSNP EALFDFGFLS* | 473 |
|  |  |
| d-BAK132[g1] |  |
| *YQKNFPS*PIC IEQQKIRDQD YEVYSLYQER KIASKAYGID TVLFSLKNFL | 526 |
| e-BAK56[g1] |  |
| *YQNNFPA*PIC ISSKQIRQQD YEVYSIYQEC KLASRIHGID SVLFSLKNFL | 525 |
| e-BAK56[g1] |  |
| YMRNFPGPQC IESTSIRQQD YEVYSIYQEC KLASKTYGID SVLFSLKNFL | 523 |

Seventeen plasma samples from African hunters were tested for binding to 37 peptides located in the SUvar domain (Supplementary Table 5). The summary graph shows positive responses (Δ_OD_, y-axis) plotted against peptides identified by the position of their first aa (x-axis). Plasma samples are identified by a color code corresponding to the genotype(s) of the infecting strains (blue: infected with a GI strain, red: infected with a GII strain, purple: infected by strains of both genotypes [1]). Left, peptides spanning GI SUvar; right, peptides spanning GII SUvar; the RBDj region is indicated by the lighter color. Detailed binding activity is shown on the aligned SU sequences; the RBDj sequence is highlighted in italic characters; the sequence covered by peptides is highlighted by grey background. Recognized peptides are underscored and designated by the same letters as those used in panel A. Reactive plasma samples are indicated above the sequence and colored according to the genotype(s) of the infecting strains. Six plasma samples from SFV-infected individuals reacted against seven peptides located in the RBDj region (BAD551, BAK55, BAK56, BAK74, BAK82, and BAK132) and two samples reacted against a peptide located in the RBD1 or RBD2 subdomains (BAD468 and BAK56, respectively). Plasma antibody binding to the peptides was not genotype-specific. For example, sample BAK56 reacted against peptide b (399W-K418) from the GI-D468 and GII-K74 strains.

1. Lambert C, Couteaudier M, Gouzil J, Richard L, Montange T, Betsem E, et al. Potent neutralizing antibodies in humans infected with zoonotic simian foamy viruses target conserved epitopes located in the dimorphic domain of the surface envelope protein. PLoS Pathog. 2018;14:e1007293.
