# Supplementary material for: Neutralization of zoonotic retroviruses by human antibodies: Genotype-specific epitopes within the receptor-binding domain from simian foamy virus
Source: PLoS Pathog. 2023 Apr 24;19(4):e1011339. doi: 10.1371/journal.ppat.1011339 (PMC10159361; doi:10.1371/journal.ppat.1011339)
Supplement: S8 Fig — HT1080 cells were incubated with SU and the bound protein detected by staining with an anti-mouse Fc antibody and flow cytometry analysis. A. The gating strategy of viable single cells and staining intensity are shown for GIISU added at three concentrations. Levels of bound GIISU are expressed as the ratio of the MFI from SU-treated cells to the MFI of untreated cells. B. To compare the binding capacity of the SU, staining was performed at three doses, the MFI ratios plotted as a function of SU concentration, and the area under the curve (AUC) calculated. Shaded regions represent the AUC. Data from five independent experiments performed with WT SU are presented as the mean and standard error. CISU bound at higher levels than GIISU. C. The treated and mutated SU were tested for binding to susceptible cells and staining levels were normalized to that of the WT SU included in every experiment. The graph shows lower staining by GIIΔRBDj than GIISU for one representative experiment. (DOCX) [file ppat.1011339.s013.docx]

## S8 Fig. Recombinant SU bind to susceptible cells

HT1080 cells were incubated with SU and the bound protein detected by staining with an anti-mouse Fc antibody and flow cytometry analysis. A. The gating strategy of viable single cells and staining intensity are shown for ^GII^SU added at three concentrations. Levels of bound ^GII^SU are expressed as the ratio of the MFI from SU-treated cells to the MFI of untreated cells. B. To compare the binding capacity of the SU, staining was performed at three doses, the MFI ratios plotted as a function of SU concentration, and the area under the curve (AUC) calculated. Shaded regions represent the AUC. Data from five independent experiments performed with WT SU are presented as the mean and standard error. ^CI^SU bound at higher levels than ^GII^SU. C. The treated and mutated SU were tested for binding to susceptible cells and staining levels were normalized to that of the WT SU included in every experiment. The graph shows lower staining by ^GII^ΔRBDj than ^GII^SU.
