# Supplementary material for: Neutralization of zoonotic retroviruses by human antibodies: Genotype-specific epitopes within the receptor-binding domain from simian foamy virus
Source: PLoS Pathog. 2023 Apr 24;19(4):e1011339. doi: 10.1371/journal.ppat.1011339 (PMC10159361; doi:10.1371/journal.ppat.1011339)
Supplement: S9 Fig — The SUvar protein sequences from the SFVggo strains circulating in Central Africa and CI-PFV were aligned. We included all available sequences: 10 genotype II sequences (nine zoonotic strains and one animal strain [11]) and 15 genotype I sequences (14 zoonotic strains and one animal strain [11]). Identical residues are indicated by dots, the background colors correspond to the physical properties (rasmol color code). Black squares indicate the identified epitopic regions: L2 (A), 345–353 loop and N7’ (B), L3 (C), L4 and E485 (D). Within each genotype, we observed identical sequences or conservative aa changes. The only exception was an N351/D polymorphism in the 345–353 loop from GI that may alter the expression of N7 strain. (DOCX) [file ppat.1011339.s014.docx]

## S9 Fig. Sequences from gorilla SFV strains circulating in Central Africa are conserved in the epitopic regions targeted by nAbs


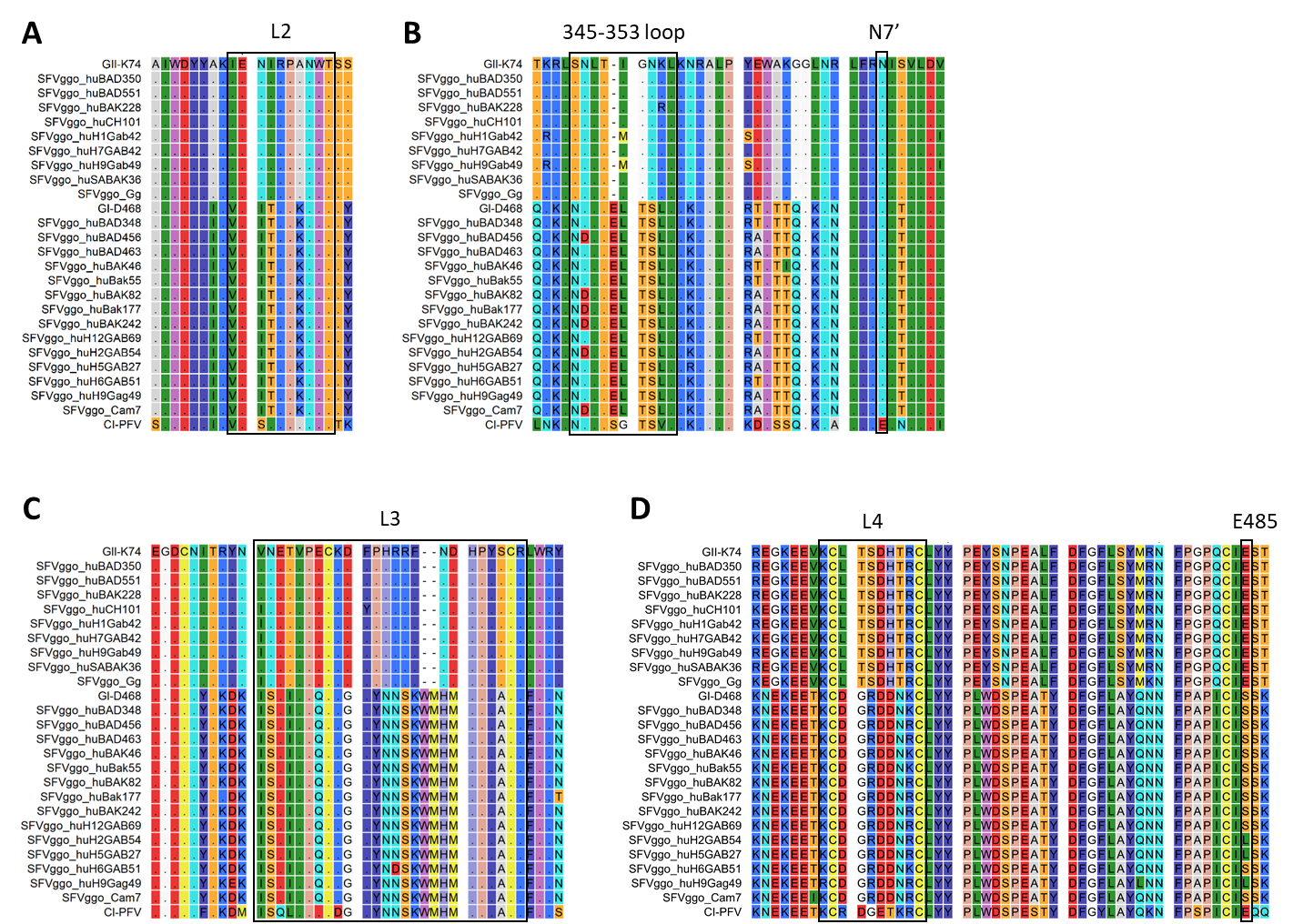


The SUvar protein sequences from the SFVggo strains circulating in Central Africa and CI-PFV were aligned. We included all available sequences: 10 genotype II sequences (nine zoonotic strains and one animal strain [1]) and 15 genotype I sequences (14 zoonotic strains and one animal strain [1]). Identical residues are indicated by dots, the background colors correspond to the physical properties (rasmol color code). Black squares indicate the identified epitopic regions: L2 (A), 345-353 loop and N7’ (B), L3 (C), L4 and E485 (D). Within each genotype, we observed identical sequences or conservative aa changes. The only exception was an N351/D polymorphism in the 345-353 loop from GI that may alter the expression of N7 strain.

1. Richard L, Rua R, Betsem E, Mouinga-Ondeme A, Kazanji M, Leroy E, et al. Cocirculation of two *env* molecular variants, of possible recombinant origin, in gorilla and chimpanzee simian foamy virus strains from Central Africa. J Virol. 2015;89:12480-91.
